# Real-time observation of dynamic structure of liquid-vapor interface at nanometer resolution in electron irradiated sodium chloride crystals

**DOI:** 10.1038/s41598-020-65274-9

**Published:** 2020-05-25

**Authors:** Amy Ren, David Lu, Edward Wong, Matthew R. Hauwiller, A. Paul Alivisatos, Gang Ren

**Affiliations:** 1 The Molecular Foundry, Lawrence Berkeley National Laboratory, Berkeley, CA 94720 USA; 2 The Department of Physics, University of California Santa Barbara, Santa Barbara, CA 93106 USA; 3 The Department of Chemistry, Brown University, Providence, RI 02912 USA; 4 Materials Sciences Division, Lawrence Berkeley National Laboratory, Berkeley, CA 94720 USA; 5 Department of Chemistry, University of California, Berkeley, CA 94720 USA; 6 Department of Materials Science, University of California, Berkeley, CA 94720 USA; 7 Kavli Energy NanoScience Institute, University of California, Berkeley, CA 94720 USA

**Keywords:** Nanofluidics, Nanoscale materials, Techniques and instrumentation, Techniques and instrumentation, Structure of solids and liquids

## Abstract

The dynamics and structure of the liquid and vapor interface has remained elusive for decades due to the lack of an effective tool for directly visualization beyond micrometer resolution. Here, we designed a simple liquid-cell for encapsulating the liquid state of sodium for transmission electron microscopic (TEM) observation. The real-time dynamic structure of the liquid-vapor interface was imaged and videoed by TEM on the sample of electron irradiated sodium chloride (NaCl) crystals, a well-studied sample with low melting temperature and quantum super-shells of clusters. The nanometer resolution images exhibit the fine structures of the capillary waves, composed of first-time observed three zones of structures and features, *i.e*. flexible nanoscale fibers, nanoparticles/clusters, and a low-pressure area that sucks the nanoparticles from the liquid to the interface. Although the phenomenons were observed based on irradiated NaCl crystals, the similarities of the phenomenons to predictions suggest our real-time ovserved  dynamic structure might be useful in validating long-debated theoretical models of the liquid-vapor interface, and enhancing our knowledge in understanding the non-equilibrium thermodynamics of the liquid-vapor interface to benefit future engineering designs in microfluidics.

## Introduction

Since Gibbs’ fundamental works on thermodynamics a century ago^1^, many models have been proposed to describe the dynamic structure of the interface between liquid and vapor, which is crucial for our understanding of the important processes in microfluidics, microbiology, and cooling systems for microelectronics^2,3^. Due to the lack of tools for directly imaging of the interface at nanometer resolution, the study is significantly more hindered than that of the solid state. As a result, molecular dynamics (MD) simulations are often used to study the dynamics of the liquid-vapor interface.

Three theoretical models have been proposed to describe the dynamic structure of the liquid-vapor interface. (i) A zero-width capillary-wave model, which is a  thin-layer interface model that was developed by Gibbs based on the fundamental theory of thermodynamics, laterly refined by Buff *et al*.^4^. In this model, the interface is treated as capillary waves created under surface tension excited by Brownian motion^5^. (ii) A nonzero-width capillary-wave model, in which the interface has high dynamics and fluctuations within a width that depended on the environments proposed by Weeks *et al*.^6^ (iii) A nonzero-width bilayer capillary-wave model^5^. In this model, the interface was proposed as a non-equilibrium thermodynamic interface, in which the above nonzero-width capillary-wave model was added with a vapor free-path zone. In this zone, the molecules moving towards the liquid surface differed in their velocities from those moving away from the liquid. The highly fluctuating and dynamic capillary wave zone has a width in the order of 10 molecular diameters located on the surface region^5,7^.

Untill recently, few experiments were conducted to study the interface and validate the theoretical models. For examples, X-ray reflectivity measurements revealed that the liquid-vapor interface exhibits the intrinsic roughness of the surface^8^, and the capillary waves drastically fluctuate on the surface of water^9^, as supporting to the nonzero-width capillary-wave model. High-speed synchrotron X-rays are also used to image the defect and molten pool dynamics *in situ* at hundred micrometer resolution^10,11^. Nevertheless, the experiments are insufficient to provide the detailed structure with direct imaging of the interface at the nanometer resolution in order to validate the interface models.

Although transmission electron microscopy (TEM) has the capability to image  hard materials at atomic resolution, the TEM vacuum column limits the imaging of liquid and vapor samples due to evaporation. To overcome this weakness, scientists recently developed a liquid-cell to encapsulate the liquid, sealing the liquid sample within a small and thin chamber^12,13^. The sealing materials must be strong enough to encapsulate the liquid under the TEM vacuum and sufficiently transparent to allow TEM beam penetration through for imaging the liquid. The materials often used to observe the chemical reaction and nanoparticle dynamics in liquid include graphene^12^ and silicon nitride^13^.

Considering that the dynamic structure of the liquid-vapor interface has never been successfully imaged by TEM, we designed a simple and highly efficient encapsulation technique for real-time TEM imaging of the dynamic structure of the liquid-vapor interface at nanometer resolution. The sample used was liquified sodium (Na) obtained from the electron-irradiated NaCl. The motivations of choosing this sample were that, i) The electron irradiated NaCl crystals has been well-studied decades ago^14–16^; ii) The Na clusters could be generated by the irradiation^17^; iii) The low molting temperature of Na clusters can be easily achieved within TEM column^18^; iv) The behaviors of molten Na clusters have been well studied by other techniques, such as differential scanning calorimetry (DSC)^19^ and wide angle X-ray scattering (WAXS)^20^; v) Quantum phenomenon have been observed in the clusters of sodium atom^21–24^; vi) The phenomenon of electron irradiated NaCl is related to the habitable, possibly even habited, planets searching in space, indicated by factors such as the surface color of the ocean on Europa^25–27^; Under these considerations, TEM images of irradiated NaCl crystals could provide experimental evidence to understand the complexity of the nanoscale clusters and validate the theoretical models of the liquid-vapor interface.

## Materials and Methods

### Assembly and evaluation of the specimen

**Figure 1:**
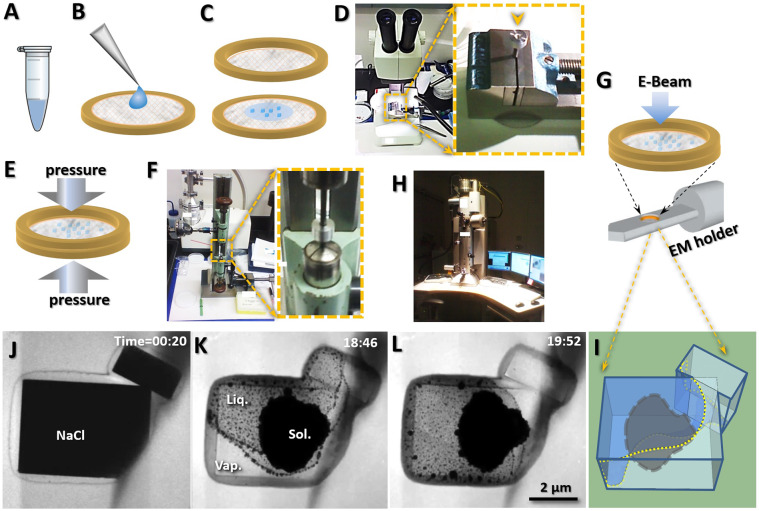
Assembly of the specimen for imaging liquid by a transmission electron microscope (TEM) Schematics of the assembled liquid-cell/micro chamber, in which **(A)** a saturated solution of sodium chloride (NaCl) was loaded onto (**B**) a Formvar plastic film that was pre-coated on a 200-mesh TEM grid. (**C)** Expanding the liquid solution to generate microscale crystals. The crystals were then sandwiched between two Formvar plastic films. (**D**) Under a light microscope, the sample was loaded on a grid and then sandwiched by aligning the grids on a washer. (**E**) The grids were subjected to physical pressure to ensure the crystals were sandwiched tightly. (**F**) The equipment used for compressing the grids under a controlled pressure. (**G**) A Zeiss Libra 120 TEM used for real-time video acquisition. (**H**) The grids were mounted on a regular TEM holder and examined by electron beam passing through the samples. **(J)** Representative TEM image of a chamber sealed two adhered rectangular NaCl crystals. **(K,L)** Two representative TEM images of electron beam irradiated NaCl for observing the liquid, vapor and clusters of nanoparticles. **(I)** Schematics of the three-dimensional (3D) shape of the liquid fluid within a chamber. Fig. A, B, C, G, E were prepared by Microsoft Office 10.0, Fig. I was drawn by SKETCHUP software (https://www.sketchup.com/), and Fig. J, K and L were the frames acquired with an OriusSC2006 CCD camera by GATAN Digital Micrograph.

The liquid-cell/micro-chamber was produced by sealing the sample between two Formvar plastic films. A NaCl sample (~99.5%) containing ~0.5% calcium silicate (MORTON table salt, Morton Salt Inc., Chicago, IL, USA) was used for the experiment, and a pure NaCl control (~99.999%, Sigma-Aldrich Co., St. Louis, MO, USA; ID: 450006, CAS: 7647-14-5) was used as a control to identify the role of the calcium silicate in the chamber. After dissolving 20 g of each sample in 40 mL of deionized water at room temperature, a ~1 mL aliquot of the saturation solution was collected from the surface into a 1 mL vial after centrifuged at 13,000 rpm for 5 min to separate the undissolved salt crystals from the solution. The saturated solution was then used for experimentation (Fig. 1A).

An aliquot of the ~0.35 μL saturated NaCl solution was pipetted onto the center of a 200-mesh TEM copper pre-coated with a thin Formvar film (Cu-200F, Pacific Grid-Tech, San Francisco, CA, USA) (Fig. 1B). The second Formvar-coated 200-mesh TEM copper grid face-to-face was touched to the solution surface and expanded it to evaporate the solution quickly, generating micron-sized NaCl crystals (Fig. 1C), and then aligned under a light microscope (Fig. 1D). The aligned grids were submitted for compression under a pressure of 12 lb/in^2^ (~0.8 atm) for ~30 s (Fig. 1E,F). Prior to the removal of the compressive force, the excess solution surrounding the edges of the grids was blotted by filter paper, and then coated with a thin layer of vacuum grease to protect the aligned grids and its containing chamber.

### Image and video acquisition

The TEM grids were mounted on a Gatan 626 TEM holder (Fig. 1G). The sample was examined using a Zeiss Libra 120 kV TEM (Fig. 1H). The microscope was operated under 120 kV high-tension with a 20-eV energy filter. The frames were acquired with a 1024 × 1024-pixel OriusSC2006 CCD camera using Gatan Digital Micrograph software. Real-time videos were screen-recorded with Virtual Dub (build 348071 released by Avey Lee) at 2–100 frames per second (fps). After recording, the software of Movie Studio Platinum version 13.0 was used to add scale bars and timers to each video based on the magnifications and frame rates, respectively.

### Irradiating and melting of NaCl crystals by electron beam

A high-intensity electron beam was used to melt NaCl crystals with diameters of 1–5 μm (Fig. 1J) under a magnification of ~4,000–40,000 times. While heating the crystals, the crystals began to shrink slightly and then quickly transformed into a free-flowing liquid (Fig. 1K). Rather than quickly evaporating under the high-vacuum column, the free-flowing liquid coexisted with the solid and vapor in the chamber, which lasted for several minutes to over one hour (Fig. 1L). The illumination angles were set to ~1.0 mrad or higher to initially melt the crystals. After a sufficient portion of the crystals was liquefied, the illumination angles were decreased to ~0.2–0.8 mrad to maintain a stable flow of fluid within the chamber (Fig. 1I).

### Statistical analysis of the particle motion

To quantitatively evaluate the particle motion, particle displacements and speeds were calculated and plotted. Videos were split into individual frames (in.tiff format) using the Adobe Photoshop 2015 software. Individual frames were then converted to the MRC image format with the *proc2d* software in the EMAN software package^28^. Next, the MRC images were stacked into a single file using the *newstack* software and viewed with *eTOMO* in the IMOD software package^29^. After manually tracking the positions of each targeted particle against the markers in each frame, the files containing all of the particle center coordinates at various times were saved and converted into an ASCII file. After being aligned to a common reference, the coordinates were used to calculate and graph the particle mean squared displacement (MSD) values. To calculate the MSD, the trajectory of each representative particle was recorded. The coordinates $({x}_{i},\,{y}_{i})$ of each particle were measured in sequential time periods (${t}_{i}$) with a step of $(\delta t)$ (*i.e*., one second in our measurement). The squared displacement ($S{D}_{i}$) within a time period between ${t}_{i}$ and ${t}_{i}$ + $\delta t$ was calculated as $S{D}_{i}={({x}_{i+1}-{x}_{i})}^{2}+{({y}_{i+1}-{y}_{i})}^{2}$. The MSD was calculated as $MSD=\frac{1}{n}\mathop{\sum }\limits_{i=1}^{n}S{D}_{i}$^30–32^, and the MSD as a function of a series of time periods was plotted with MICROSOFT EXCEL.

## Results

The fluid was generated by the electron beam irradiated NaCl crystals (with 0.5% calcium silicate), which were sandwiched between Formvar plastic films coated on two TEM grids (Fig. 1A–F). To prevent leakage, the circumference of the TEM grids was sealed by vacuum grease. The sample was mounted on a regular TEM holder and examined by a Zeiss Libra 120 TEM under room temperature (Fig. 1G–J).

**Figure 2:**
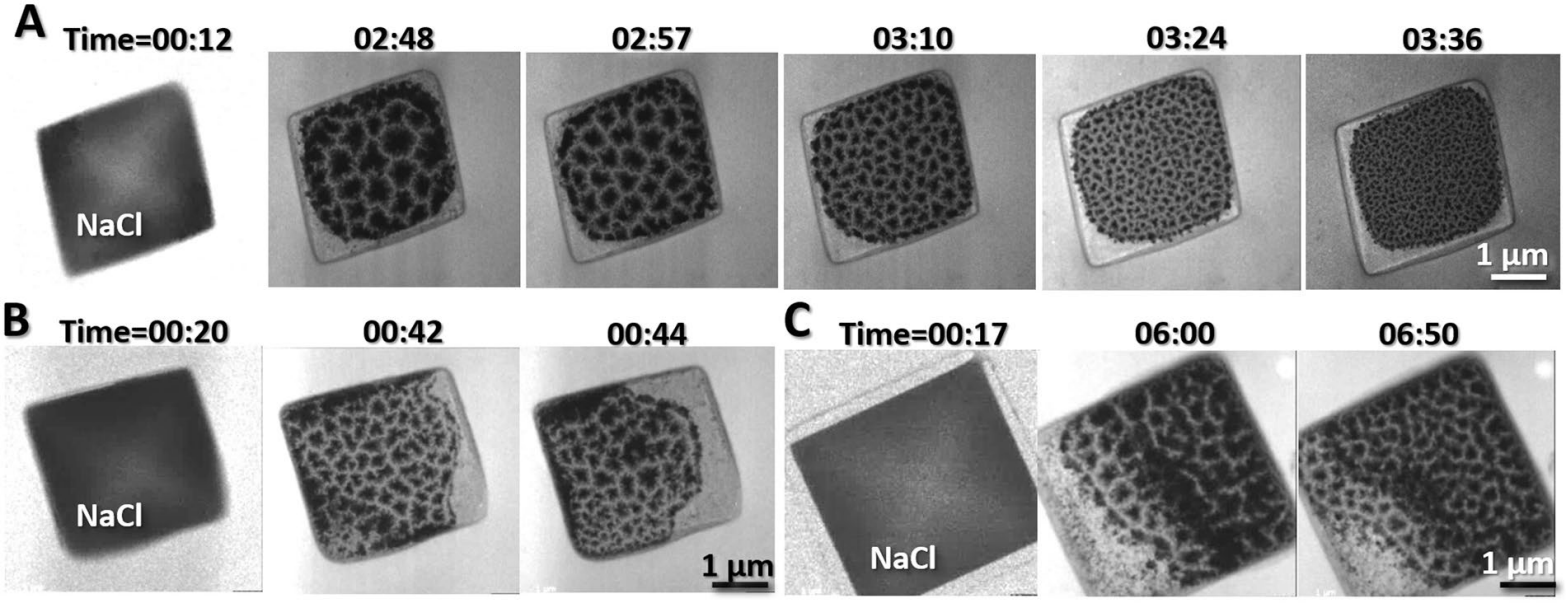
Reproducibility for observation of liquid flow on three NaCl crystals. (**A**) A NaCl crystal was heated and irradiated by the electron beam. By increasing the illumination intensity, electrons impacting the crystals resulted in the crystals shrinking, melting and suddenly transitioning into a free-flowing liquid, which filled the chamber. Under a fixed illumination angle, the formation, movement, and flow of the coexisting solid, liquid, and vapor phases were observed. **(B,D)** By repeating the above experiment on other two NaCl crystals, similar phenomenon can be observed. Figs. were the frames acquired with an OriusSC2006 CCD camera by GATAN Digital Micrograph.

With the electron beam focused on the crystals, the irradiated crystals shrank, and then turned into fluid (Figs. 1J–L and 2 and Supplementary Video 1). The fluid was mixed with three phenotypes, *i.e*. clusters of solid particles, liquid and vapor. These three objects can be distinguished based on their distinct contrasts, in which the solid-state particles exhibited the poorest transparency, while the vapor-state presented the highest transparency (Figs. 1K,L and 2). The materials of those objects have been well  studied before. For instance, the time-resolved X-ray analysis on the electron irradiated NaCl using TEM energy dispersive spectrometer showed the chlorine (Cl) evaporated as vapor and dispersed into the vacuum as gas, while the Na deposited as solid and liquid diffused to the surrounding^17^. The result was consistent to early studies, such as that the melting temperatures of the clusters of irradiated Na and NaCl were ~30–40% below the molting temperature of Na (98 °C) based on the measurements from various techniques, including heat capacities^18,33^, differential scanning calorimetry (DSC) and atomic force microscopy (AFM)^19^, X-ray diffraction^20^, molecular dynamics simulations^34^, Raman spectra^16^, and optical-absorption spectroscopy^14,15^. The low melting temperature of clusters enable us to image the fluid encapsulated by Formvar film (the melting temperature of the Formvar film is ~103–113 °C).

**Figure 3:**
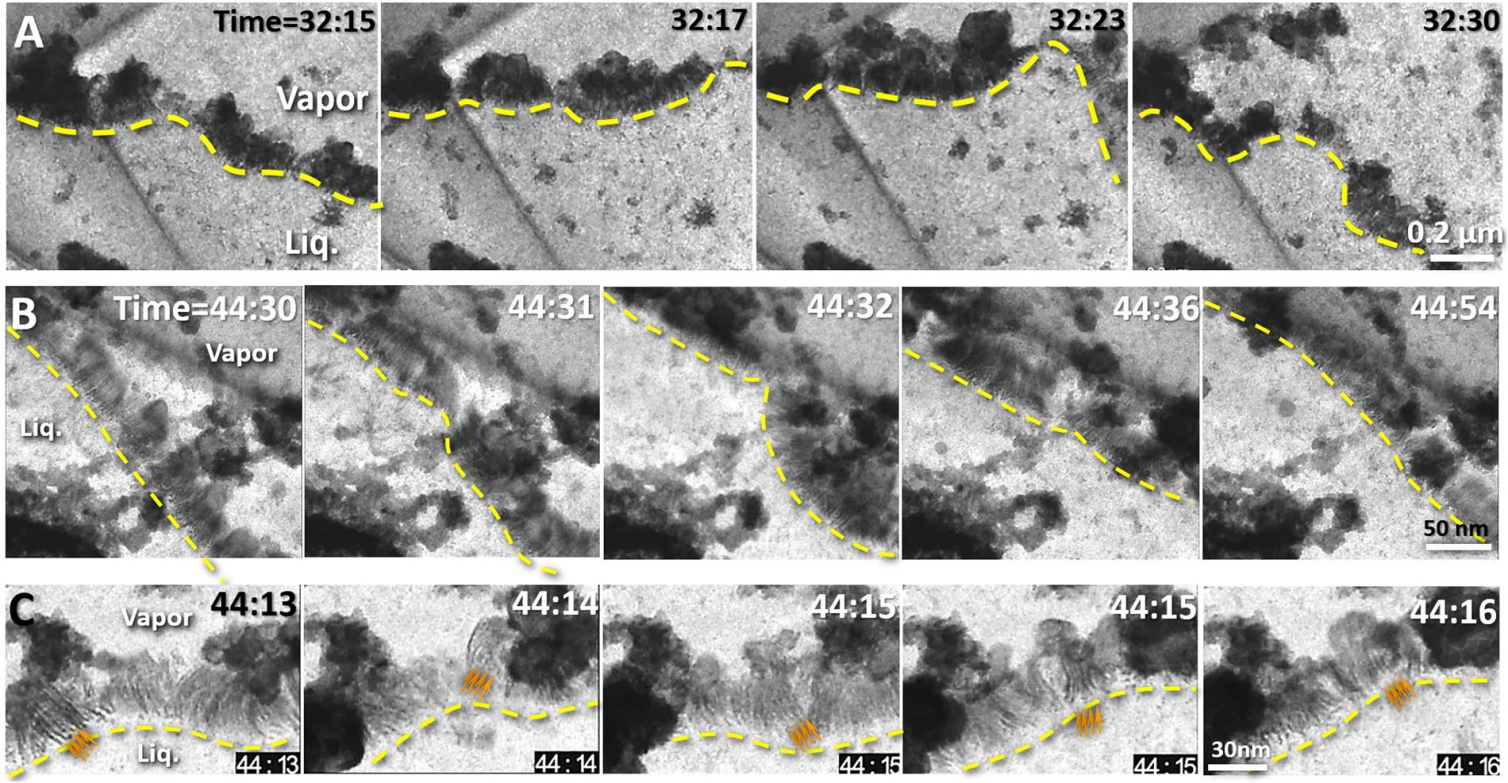
The fluctuation and dynamic structure of the liquid-vapor interface (**A**) Sequential movie images showing the liquid-vapor interfaces over time. The dashed yellow lines outline the shape of the interface, which fluctuated significantly over short intervals of time. (**B**) Sequential movie images showing another liquid-vapor interface over time at a higher magnification. The dashed line outlines the shape of the interface, which fluctuated significantly over short intervals of time. (**C**) Magnified images of a third liquid-vapor interface at an even higher magnification. The arrows indicate the structural details of the liquid-vapor interface. Figs. were the frames acquired with an OriusSC2006 CCD camera by GATAN Digital Micrograph.

Under an illumination of dose rate ~3,000 e/nm^2^/s, the irradiated sample exhibited a rapidly fluctuating fluid boundary, filled with clusters of nanoparticles with diameters of ~50–200 nm (yellow lines in Fig. 3A and Supplementary Fig. 1 and Supplementary Video 2). One side area of the boundary is  more transparent, containing more aggressive motion of the nanoparticles. This area was filled with vapor. In contrast, the opposite side of the boundary was less transparent, filled with nanoparticle moving less aggressively. This area was as filled with liquid. The fluctuating boundary, as the liquid-vapor interface, has a speed of over 300 nm/s (Fig. 3, Supplementary Fig. 1 and Supplementary Video 2). In a low magnification, the fluctuating boundaries could be observed forming a closed loop shape within the chamber (Fig. 1K,L), in which two portions of the boundaries were moving independently, and sometimes could cross into and pass through one  another, forming an “8” shape (Supplementary Video 2). Occasionally, the middle of the boundary extruded a smaller loop, likely caused by a concave surface of the liquid. Mover, the boundaries never exceeded beyond the chamber edges but can pass over a central “island” (a roundish solid particle clusters in diameter of ~2 μm) freely (Fig. 1J–L and Supplementary Video 2). The above phenomenon suggested the liquid surface within the chamber has a 3D shape and the observed boundaries were that of the liquid surface crossing with the chamber walls **(**Fig. 1I**)**. The observation of the liquid-vapor interface fluctuation was replicable on different crystals (Fig. 2), and the fluctuation phenomenon can be observed for over one hour (Supplementary Videos 1 and 2). Since the edge of the liquid surface against the chamber wall exhibited high image contrast with structural features, the structure of this boundary was focused on in this study.

**Figure 4:**
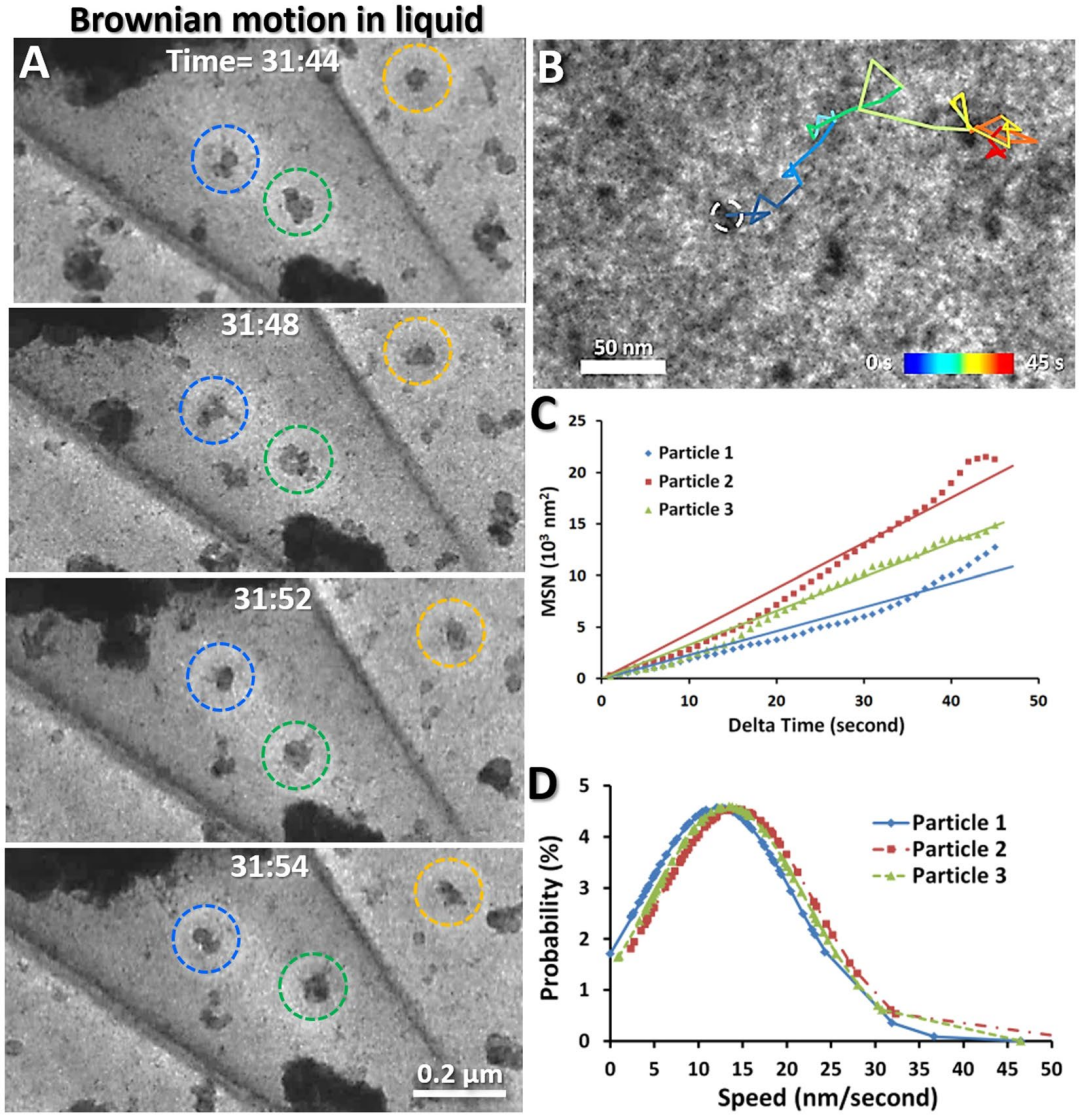
Brownian motion of nanoparticles in liquid (**A**) Snapshot images show three representative particles in liquid undergoing random movement. (**B**) The colored segments showed the trajectory of a representative particle (~15 nm in diameter) in liquid. Each segment corresponds to a different time interval within the 45 s frame. (**C**) Plots of the mean square displacements (MSD) against the time intervals, *Δ*t (40 s), in which the MSD of three representative particles were fitted with a least-squares regression line to characterize the Brownian motion. (**D**) Particle speed probability distributions of above three representative particles were computed and fitted. Figs. A and B were the frames acquired with an OriusSC2006 CCD camera by GATAN Digital Micrograph, Figs. C and D were prepared by MICROSOFT EXCEL.

**Figure 5:**
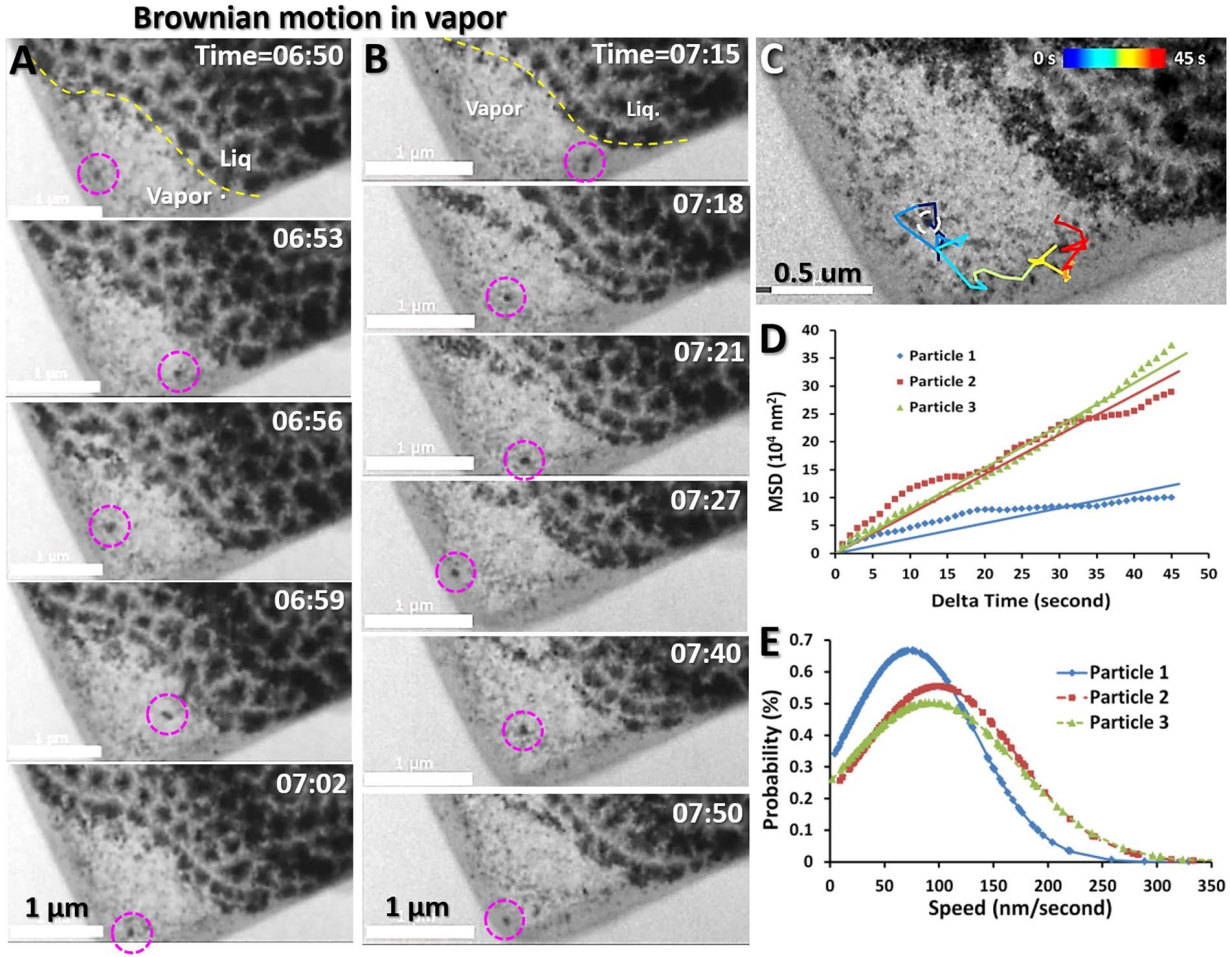
Brownian motion of nanoparticles in vapor (**A**,**B**) Sequential movie images showing two representative solid particles exhibiting random motion in the vapor phase. The yellow dashed line in the first image in each column outlines the liquid-vapor boundary, and the particles in the dashed circles illustrate the changing locations over short intervals of time. (**C**) A representative trajectory of one selected particle (~50 nm in diameter). The trajectory was shown in colored segments within the 45 s frame. (**D**) MSD-*Δ*t plots of the three particles within 45 s are fitted with a least-squared regression line to show the linear relationship between displacement and time indicated as Brownian motion. (**E**) Speed probability distributions of the three particles were computed and fitted. Figs. A, B and C were the frames acquired with an OriusSC2006 CCD camera by GATAN Digital Micrograph, Figs. D and E were prepared by MICROSOFT EXCEL.

In both liquid and vapor, large amounts of nanoparticle clusters were observed undergoing random motion (Supplementary Video 2). To quantitatively identify the motion, we targeted three representative nanoparticles in each side and tracked their trajectories from video frames (Figs. 4 and 5 and Supplementary Videos 3 and 4) to calculate the particle travel distance, instantaneous speeds, and mean square displacements (MSD). Three representative nanoparticles in liquid showed a maximal displacement distance (from the origin) of over ~0.16 μm within 60 s (Fig. 4A,B and Supplementary Video 3). The MSD exhibited a nearly linear association for different time intervals, *Δ*t, from 1 s to 40 s (Fig. 4B), suggesting that the particle motions satisfy the criteria for Brownian motion. The histograms of the instantaneous speeds of each particle resemble Maxwell curves with a relatively consistent peak speed at ~14 nm/s (Fig. 4C).

Particles in vapor exhibited more aggressive motion than particles in liquid. By tracking the trajectories of three representative nanoparticles in vapor, the result showed the particles travelled a maximal displacement distance (from the origin) of over ~1.4 μm within 60 s (Fig. 5A–C and Supplementary Video 4), which was about 9 times greater than that of the particles in the liquid. The MSD-*Δ*t plot also exhibited a nearly linear association for 45 s (Fig. 5D), indicative of Brownian motion. Similarly, the histograms of the instantaneous speeds also resemble Maxwell curves with a relatively similar peak speed of ~90–120 nm/s (Fig. 5E), which were approximately 6–9 times greater than that in liquid. The distinct differences in the maximal travel distance and the peak speed confirmed that these two groups of particles travelled in different mediums, *i.e*. the particles with lower travel distances and lower average speeds were likely embedded in liquid, whereas the particles with higher travel distances and speeds support that the particles were likely surrounded by vapor.

**Figure 6:**
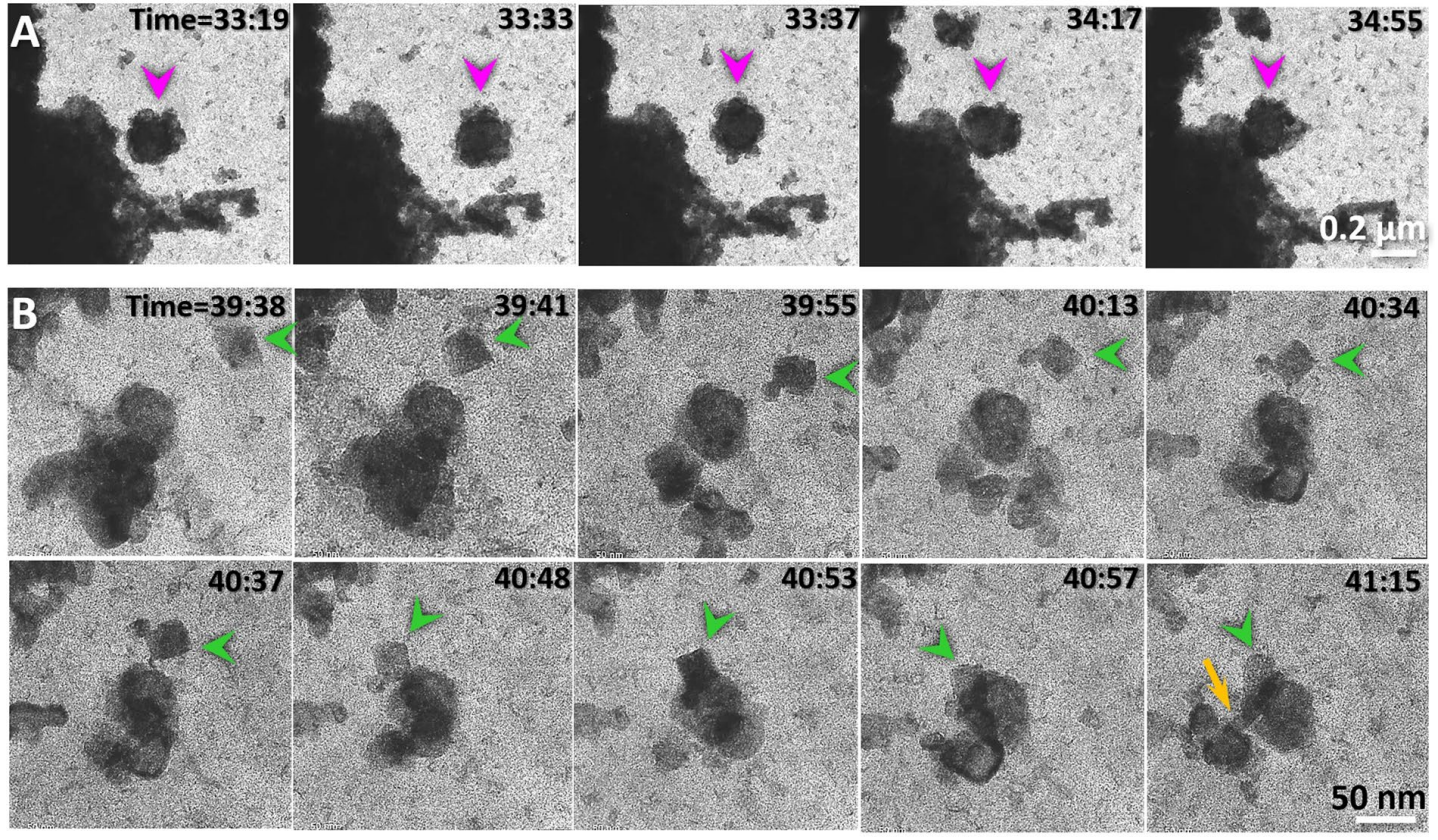
Nanoparticle interactions. (**A**) In liquid, an ~200 nm globular particle (indicated by purple arrows) occasionally interacted with an ~1 μm cluster from random orientations. The attachment and separation between those two particles showed weak inter-particle interaction. (**B**) An ~30 nm rectangular particle (indicated by green arrows) was oriented itself within a range of ~50 nm and then interacted to an ~50 nm globular particle with a specific direction. Immediately prior to the attachment, a fiber-shaped density formed between the particles, bridging the two particles and bending them to bring them closer before the two particles merged (indicated by an orange arrow). Figs. A and B were the frames acquired with an OriusSC2006 CCD camera by GATAN Digital Micrograph.

**Figure 7:**
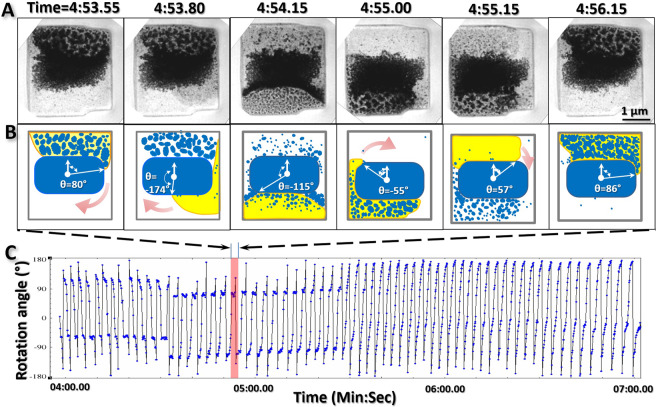
A micrometer-scale “two-cylinder motor” for liquid spinning (**A**) Six representative video snapshots within a representative spin cycle show the flow of liquid around a central large solid (~1.5 μm × ~2.5 μm) within an ~3 μm × 3 μm chamber. **(B)** Six representative illustrations of the snapshot images. The liquid is indicated in yellow, the solid particles are indicated in blue, and the front flow boundary is indicated with an orange outline. The angles of the front flow boundaries were measured and shown as the angle between two vectors. **(C)** Representative cycle of ~70 cycles observed within ~3 min. The angles of the front flow boundary are measured from each frame and plotted versus time. Fig. A was the frames acquired with an OriusSC2006 CCD camera by GATAN Digital Micrograph, Fig. B was prepared by MICROSOFT POWERPOINT, and Fig. C was prepared by MICROSOFT EXCEL.

Under electron irritation, the particles often exhibited collisions (Fig. 6A), fusion (Fig. 6B) and sublimation (Supplementary Video 5). The nanoparticle fusion was often shown in liquid through collisions from random orientations as well as through specific orientations (Fig. 6B) as reported^35^. Sublimation often occurred in vapor. Increases in sublimation frequency corresponded with more rapid fluctuation of the liquid boundary. Under a specific illumination condition, the aggressive sublimation and rapid flow of the liquid can be dynamically balanced. For example, we observed the liquid spins within the chamber for ~70 cycles in a period of ~3 second/cycle (Fig. 7 and Supplementary Video 6). In each cycle, the phenomenon of liquid flow and sublimation was similar to that in other cycles, *i.e*. the opaque liquid area containing particle clusters formed a semicircular shape stabilized against a chamber wall beside a central “island” particle. After ~1 s, the liquid began solidify and exhibited less transparency due to the increasing number of large particles (Fig. 7A,B). When the particles grew to ~300 nm in diameter, the liquid was suddenly sprayed out from the original position and pulled into the opposite half of the chamber from the same direction. The flow left the particles behind in their original positions (Supplementary Video 6). While the particles were sublimated gradually, the liquid against the opposite wall began to squeeze together. In the meantime, newly-generated particles began showing up in the liquid (Supplementary Video 6). This process is the first half-cycle of spinning. After stabilizing for ~1 s, the liquid reformed into a semicircular shape filled with regenerated opaque particles. The condition was similar to that of the liquid in the opposite side at the beginning of this cycle. This marks the beginning of the second half of this cycle (Fig. 5A,B). The spinning process in each cycle was essentially similar to each other and the subsequent cycles followed the same pattern for nearly six dozen rounds and lasted for about 4 minutes of observation until we intentionally stopped it due to having recorded sufficient video (Fig. 5C and Supplementary Video 6).

The mechanism of the above liquid spinning phenomenon can be interpreted as a microscale dual-cylinder motor, in which the spinning of the liquid was driven by the electron beam through the processes of nanoparticle generation and sublimation. In brief, the particles act as the source for absorption of the electron beam energy due to their low-transparency property. The over-heated particles quickly turned themselves into vapor via sublimation. The vapor pressure pushed the surround liquid into the opposite half side of the chamber. The continual sublimation of the rest particles generated more vapor and a higher pressure that squeezed the liquid volume, resulting an increased melting temperature of containing particles. The increased melting temperature caused the low-energy molecules/small particles to grow, displayed as the newly-generated solid particles in the liquid. The newly-generated solid particles in the liquid acted as a new source for absorption of the electron beam energy for the next half of cycle. The liquid spinning,  microscale dual cylinder motor may inspire future designs of  micrometer-scale motors.

**Figure 8:**
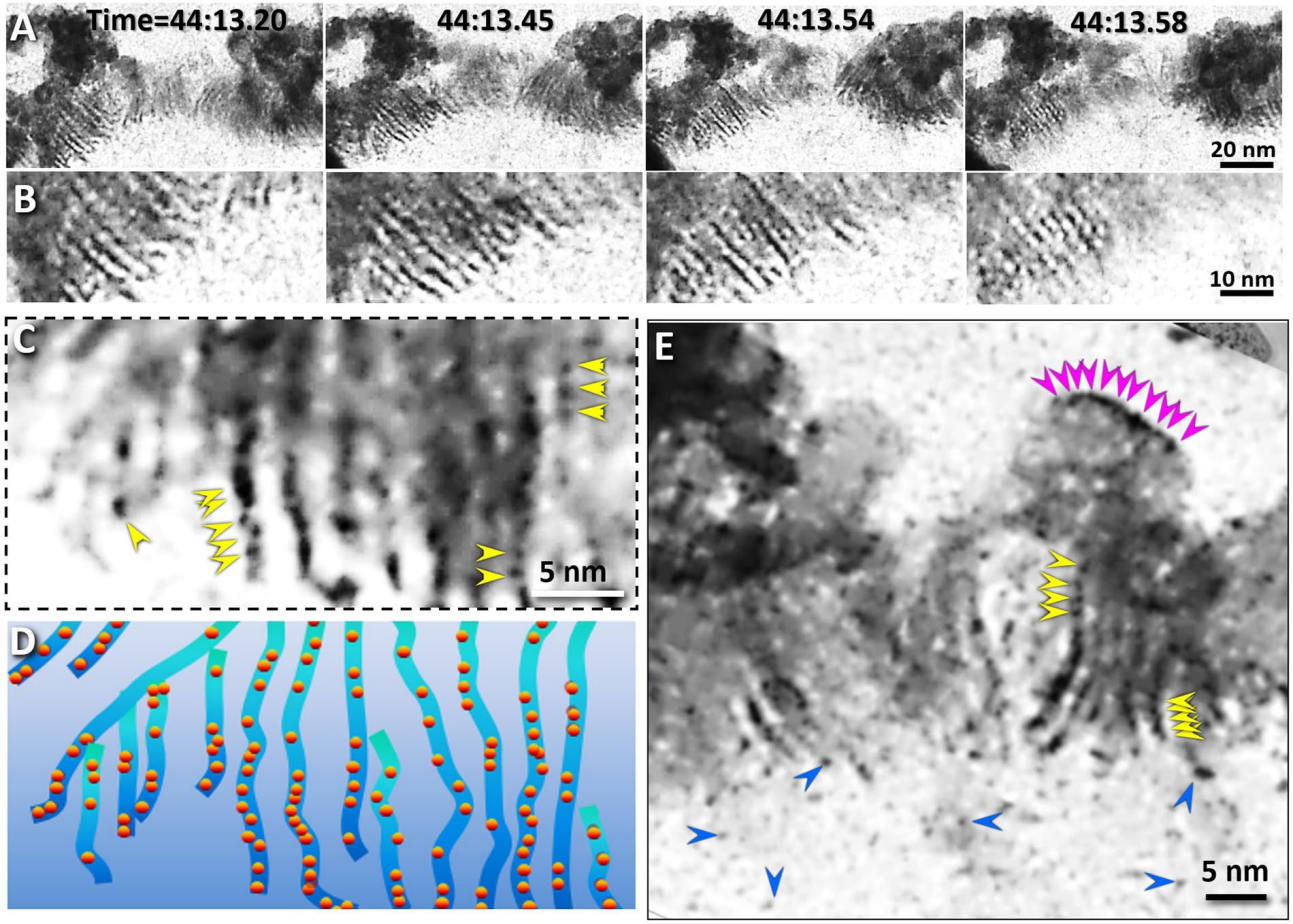
Structural details of the nanofiber zone of the liquid-vapor interface (**A**) Four snapshot images and **(B)** their zoomed-in images of the central portion of the liquid-vapor interface. The fibers had widths of ~1 nm and lengths of ~20–30 nm. **(C)** Each fiber contained several nanoparticles ~1 nm in diameter (indicated by yellow arrows) in the additional zoomed-in image of fibers. **(D)** The schematics of the fibers and their incorporated nanoparticles. (**E**) A zoomed-in image of another central portion of the interface. the ~1 nm nanoparticle located in the liquid (blue yellow arrows), within the fibers (yellow arrows) and the surface shell of the large globular particles within the nanoparticle cluster zone (magenta arrows). Figs. A, B, C and E were the frames acquired with an OriusSC2006 CCD camera by GATAN Digital Micrograph, and Fig. D was prepared by MICROSOFT POWERPOINT.

Zooming-in on the liquid-vapor boundary at a high magnification under a relatively stable fluctuation condition, the capillary waves showed detailed structural features (Fig. 8, Supplementary Figs. 2–4 and Supplementary Video 7). The most attractive feature was the liquid-vapor interface exhibiting a layer of nanometer-scale fibers, named as the nanofiber zone. The fibers extended from the bulk liquid side toward the vapor side. The fibers have a relatively uniform length of ~30 nm and a width of ~2 nm and were relatively parallel to each other with an average spatial distance of ~2–4 nm (Fig. 8C,D). The fibers oscillated frequently in response to the motions of capillary waves (Supplementary Video 7). The ends of the fibers in the bulk liquid side were relative stable in their positions. Interestingly, other than isolated tiny particles in the liquid (blue arrows indicated in Fig. 8E**)**, a chain of tiny particles in diameter of ~1 nm was visible within each fiber (yellow arrows indicated in Fig. 8C,E, and purple dots in Fig. 8D). In contrast, the opposite end of the filberts swung aggressively, and generally bent toward to large globular particles in diameter ranging from ~10 to ~300 nm in the vapor side. The clusters of those globular particles form a boundary along the liquid-vapor interface (Fig. 8E), named as the nanoparticle cluster zone. On the surface of each globular particle, the tiny particles were often observed along the vapor side of the globular particle surface (purple arrows indicated in Fig. 8E).

Based on the Supplementary Video 7, we found the nanofibers act as a nanoscale conveyor belt for transferring the materials from the bulk liquid side to the vapor side. The transferred materials included the liquid, the tiny particles within the fibers and the clusters that were transferred to the globular particles to contribute to the growth of globular particles in the cluster zone (Fig. 7f and Supplementary Video 7). Notably, once the globular particles grew to approximately 300 nm in diameter, they began to shrink, turned transparent and finally evaporated via sublimation (Supplementary Video 7). Considering the similar size of globular particles observed in the liquid in the above-mentioned spinning phenomenon, the magic number of 300 nm may reflect a balance point between the absorption rate of electron energy and sublimation temperature.

**Figure 9:**
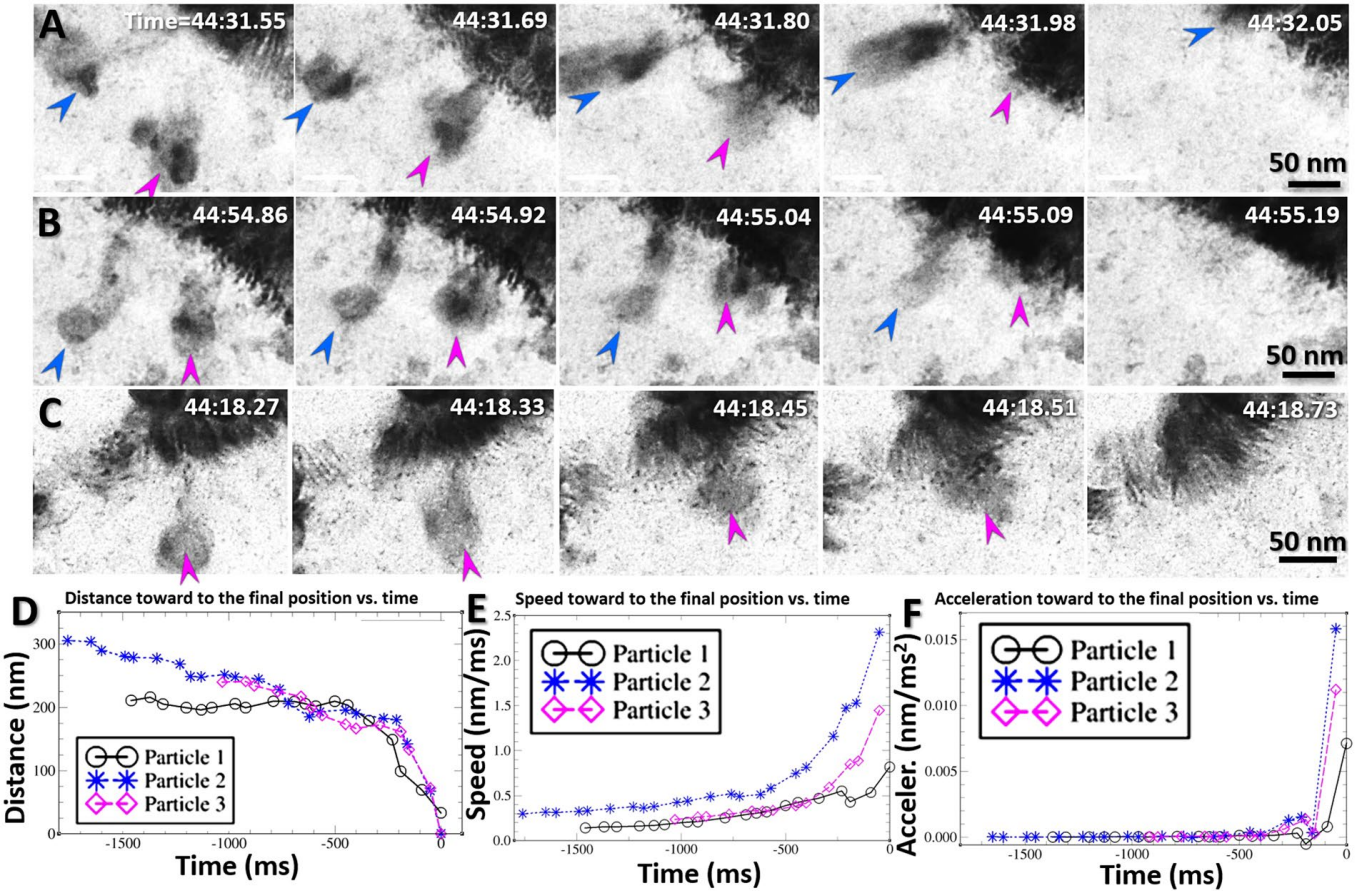
Absorption of nanoparticles within the low-pressure zone of the liquid-vapor interface (**A**) Sequential half-second snapshots from the video showing two representative particles with diameters of ~50 nm (indicated by the blue and magenta arrows) which were attracted to and then absorbed by the boundary. **(B**,**C)** Three more representative particles were moved close to and then absorbed into the liquid-vapor interface. **(D)** The distance measured between the particle and the final absorption position on the boundary of the liquid-vapor interface varied with time. Correspondingly, the time-dependent **(E)** speeds and **(F)** accelerations of these particles were also computed to confirm the sudden motion of the particles to their destinations. Figs. A, B, and C were the frames acquired with an OriusSC2006 CCD camera by GATAN Digital Micrograph, and Figs. D, E and F were prepared by MICROSOFT EXCEL.

Another unexpected finding from the Supplementary Video 7 was that, once the nanoparticles in the bulk liquid approached the interface within ~50 nm distance, they were suddenly sucked toward the nanofibers (Fig. 9A–C, Supplementary Fig. 5 and Supplementary Video 7). The measurements of the distances, velocities and accelerations of the particles confirmed this observation (Fig. 9D–F). The travel process was so aggressive (>50 nm/s) that nanoparticle shapes were elongated, as a nearby star is stretched out  just before it gets sucked into a black hole in outer space. Although, the structure did not show any abnormal feature or contrast within ~50 nm distance from the interface, this sucking phenomenon suggests a low-density gradient zone existing in the bulk liquid, as low-pressure zone. Although this zone was detected in the liquid Na, a similar low-pressure zone has been predicted in molten salt KI by MD simulation^36^, suggesting the liquid-vapor interface observed from liquid Na may be a general phenomenon of liquid.

The physics behind the trapping phenomenon is unknown. A potential mechanistic explanation was that escaped high energy molecules from liquid to vapor caused a decrease in the average kinetic energy and the local temperature near the capillary wave. The lower temperature turned some low-energy molecules in liquid into the solid phase, as presented by the nanofibers and nanoparticle clusters. The escaped molecules and solidified molecules left some empty space in liquid, which resulted a local low density in the liquid, and the low-density area generated a pressure to the nearby object. The relatively large flowing particles received more force due to its larger surface area. The force pulled the particle in the bulk liquid toward the interface as expressed as the sucking or trapping phenomenon. The more molecules escaping from the liquid surface, the lower the local temperature, the more solidifying molecules, the more liquid flowing to the interface and the greater growth of globular particles in the cluster zone. On the other hand, for the globular particle, the more energy absorbed from the electron beam, and the more aggressive the increase in particle temperature. As the nanoparticle diameter grew to ~300 nm, the temperature become high enough t for sublimation. The sublimation generated a local gas pressure, which pushed the liquid front backwards towards the interface, resulting in a dynamic balance of the interface.

**Figure 10:**
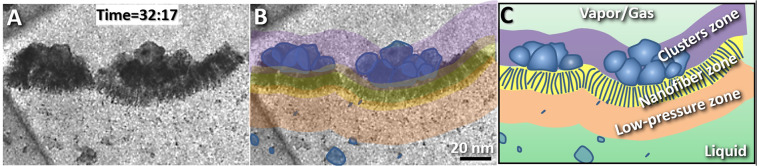
Structural model of the liquid-vapor interface (**A)** Magnified image of a boundary showing the structure of the liquid-vapor interface. **(B)** The boundary was composed of three zones with different characteristics. Yellow represents the nanofiber bundle zone, orange represents the nanoparticle trap zone, and lavender represents the nanoparticle cluster zone. The relatively large nanoparticles are colored in blue. **(C)** The schematic of the model of the liquid-vapor interface. Figs. A and B were the frames acquired with an OriusSC2006 CCD camera by GATAN Digital Micrograph, and Fig. C was prepared by MICROSOFT EXCEL.

In short, the above observations showed the capillary wave has a rough interface with a  thickness of ~300–400 nm (Fig. 10). The interface was composed of three types of structural zones (~50 nm for the vacuum zone, ~30 nm for the nanofiber zone and ~200–300 nm for the cluster zone). This model favors the nonzero-width bilayer capillary-wave models and is consistent with the asymmetric capillary-wave model^6^, in which nanofibers and rapidly-moving nanoparticles were predicted. However, our model is not consistent with the zero-width elastic capillary-wave models predicted by Gibbs^1^ and Buff^4^.

## Discussion

The TEM observation on the fluid dynamics for more than an hour benefited from the two materials used to encapsulate the liquid under the vacuum, *i.e*. Formvar double films and ~0.5% calcium silicate that mixed with the NaCl crystals. To investigate the role of those two materials, we conducted the following three experiments (Supplementary Fig. 6). i) To evaluate the role of calcium silicate, the pure NaCl crystals (~99.999%, without calcium silicate) were used to repeat the experiment. Upon melting, the fluid phenomenon appeared similar to that observed in our experiments (Fig. 2). However, the fluid phenomenon disappeared after a few minutes **(**Supplementary Fig. 2A**)**, suggesting the calcium silicate in our experiment plays a key role to prevent leakage of the liquid into TEM vacuum. ii) To evaluate the role of Formvar double films, we repeated the experiment by using a single Formvar film (adhered on a holey carbon film of TEM grid) instead of the double films. The same NaCl crystals containing ~0.5% calcium silicate was deposited on the surface of the Formvar film and subjected to  TEM imaging of the liquid. During the electron beam irradiation  of the crystals, a circular shadow was observed expanding from the irradiated crystal (Supplementary Fig. 2B, indicated by the dashed arc line). Despire observing  clusters of particles, the fluid phenomenon was never observed during evaporation. Notably, a shell of the original crystal remained in its original position during the entire process of irradiation, suggesting the calcium silicate mainly coated on the surface of the NaCl crystals (the dashed straight line in Supplementary Fig. 2A). iii) To test whether the surface of the NaCl crystal was coated with calcium silicate, the pure NaCl crystals (~99.999%, without calcium silicate) were deposited on the single Formvar film and then submitted for TEM irradiation. Upon melting the crystals, spherical droplets appeared on the surface of crystals (Supplementary Fig. 2C). Some droplets fused into larger droplets, while some small droplets jumped out from the crystal surface and then quickly burst in the background (Supplementary Fig. 2C). After the whole crystal was evaporated, no shell was left behind. This experiment confirmed the calcium silicate coated on the NaCl crystal surface as a shell, which was consistent to the original design in table salt. In table salt, the calcium silicate was often used as an anticaking agent to prevent the formation of lumps and caking. Considering the fluid phenomenon was only observed by using the double Formvar films regardless of the existence of calcium silicate, the double Formvar films played most important role to seal the liquid. However, since  leakage prevents a long imaging time necessary to observe the liquid, the calcium silicate was necessary to secure the shell for over one-hour observation of the sealed liquid.

Although our phenomenon was observed based on the liquid phase of Na, the large common features and characteristics of liquid is consistent to that from other samples. For instance, the studies of lithium chloride (LiCl) and potassium chloride (KCl) molten salts by molecular dynamics (MD) simulations predicted the clusters exhibited in the interface^37,38^, in which three types of particles were predicted near the interface, *i.e*. internal particles, surface particles, and virtual chains of particles. These results have strong similarities to our observations of liquid Na. Moreover, the MD studies of molten potassium salt (potassium iodide, KI) demonstrated that the formation of clusters of ions, separated by large spaces of vacuum, can be appreciated at the interface^36^, which supported the bilayer model^5^. The vapor free-path zone in bilayer model described the average velocity of the molecules moving towards the liquid is different than that  away from liquid^5^. These descriptions are similar to the observed vacuum zone. Moreover, the surface region of the capillary wave zone, having a width of the order of 10 molecular diameters, is highly variable as a result of thermodynamics^5,7^, which is similar to our observed nanofiber zone. Additionally, the prediction that the rapidly-moving capillary waves created by surface tension were not fully explainable by Brownian motion^5^ due to the heavy involvement of phase transitions, is also consistent with our observation. Interestingly, MD simulations studies of a completely different material system also predict similar phenomenon to our observations. For example, the MD studies of argon-like fluid suggested that the transition from liquid to vapor in the interface is locally sharp with great fluctuations^39^, similar to our observation of nanofiber zone. Surprisingly, the virtual chains of molecules near the interface were also predicted by MD simulations^40^, which is similar to our observed nanofibers and the chain of ~1 nm nanoparticles within the fibers. In MD simulations, fibers formed rough protrusions, causing capillary waves to act as a membrane^41^, which was validated by our observed nanofiber zone. The orthogonal experiment by X-ray reflectivity measurements of gas-liquid interactions also validated this prediction. The measurements suggested the surface of an ionic liquid exhibits the intrinsic roughness of the surface^8^, where a bimolecular layer drastically enhances capillary wave fluctuations on the surface of water^9^. The large similarities and consistencies of the phenomenon of the liquid-vapor interface suggest that, although our observation was based on electron irradiated NaCl, the phenomenon can reflect the general characters of liquid regardless of the materials.

## Conclusion

The real-time imaging of dynamic processes by a regular TEM holder without any special modifications provides a unique approach to enhance the capability of traditional TEM in studying reactions in liquid. The detailed dynamic structure of the liquid-vapor interface validated the theoretical models and provided a unique model. A large amount of new phenomenon may help us understand the thermodynamic principles and fluid behaviors at nanometer scale, which will benefit future microfluidic developments. The implementation of this method to encapsulate other critical materials may assist us to understand the fundamental principles behind nanoparticle interactions, nano-crystallization, metal domain generation, chemical reactions, and multiple phase transitions in future.

## Supplementary information

Supplementary Information.

Supplementary Video 1.

Supplementary Video 2.

Supplementary Video 3.

Supplementary Video 4.

Supplementary Video 5.

Supplementary Video 6.

Supplementary Video 7.
