# Supplementary material for: Real-time observation of dynamic structure of liquid-vapor interface at nanometer resolution in electron irradiated sodium chloride crystals
Source: Sci Rep. 2020 May 25;10:8596. doi: 10.1038/s41598-020-65274-9 (PMC7248077; doi:10.1038/s41598-020-65274-9)
Supplement: Supplementary file 1 — Supplementary Information. [file 41598_2020_65274_MOESM1_ESM.pdf]

Supplementary Information
for

Real-time observation of dynamic structure of liquid-vapor interface at nanometer resolution in electron irradiated sodium chloride crystals

Amy Ren[1,2], David Lu[1,3], Ed Wong [1], Matthew R. Hauwiller[4,5,6,7], A. Paul Alivisatos[4,5,6,7], Gang Ren[1,*]

[1] The Molecular Foundry, Lawrence Berkeley National Laboratory, Berkeley, CA 94720
[2] The Department of Physics, University of California Santa Barbara, CA 93106, USA,
[3] The Department of Chemistry, Brown University, Providence, RI 02912, USA,
[4] Materials Sciences Division, Lawrence Berkeley National Laboratory, Berkeley, CA 94720, USA,
[5] Department of Chemistry, [6] Department of Materials Science, and [7] Kavli Energy NanoScience Institute, University of California, Berkeley, CA 94720, USA

*Correspondence should be addressed to G. R. (gren@lbl.gov)

Supplementary Figures

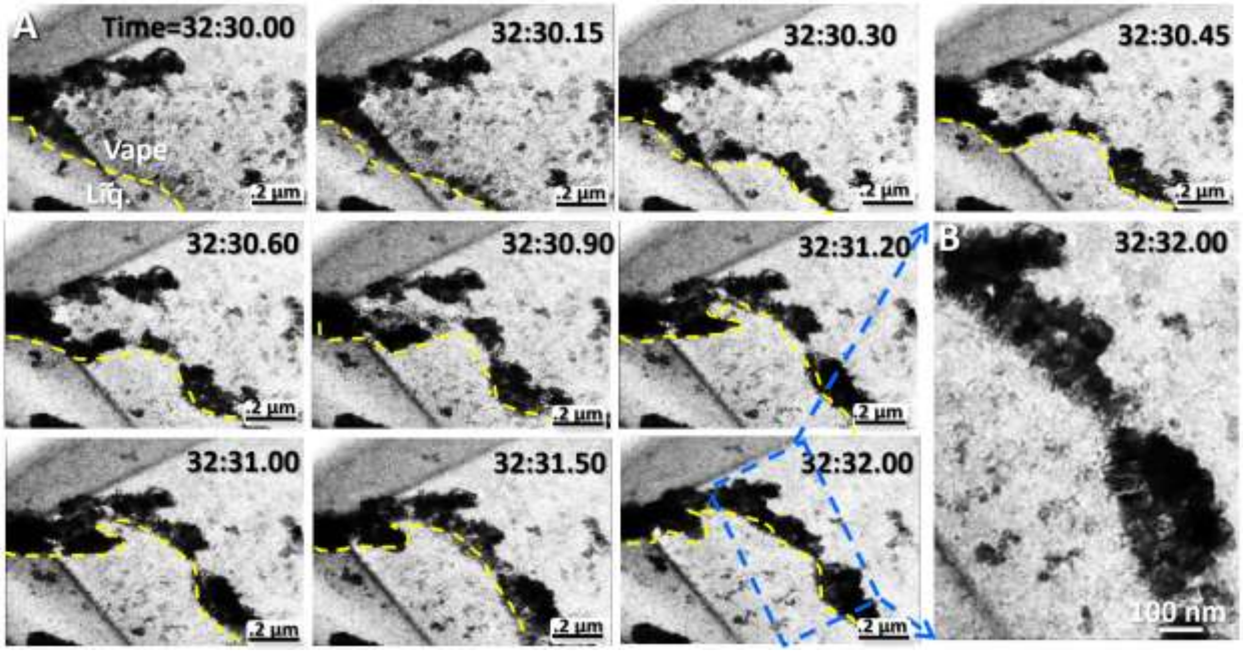

Supplementary Fig. 1 | The dynamic structures of a liquid-vapor interface. (A) Sequential movie images showing the liquid-vapor interfaces over time. The dashed line outlines the shape of the interface, which fluctuated significantly over short intervals of time. **(B)** A magnified image of a boundary showing the structure of the liquid-vapor interface. Figs. were the frames acquired with an OriusSC2006 CCD camera by GATAN Digital Micrograph.

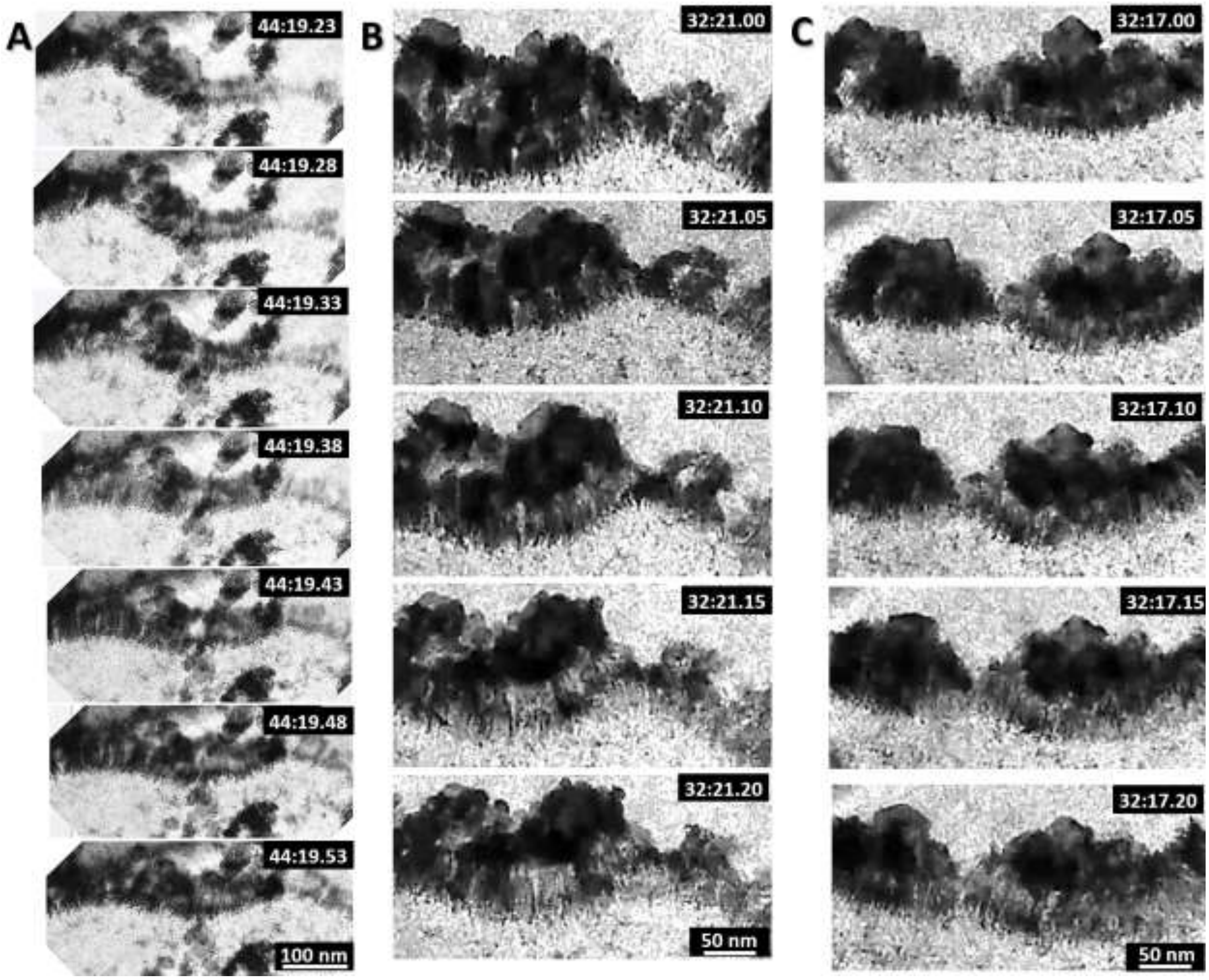

Supplementary Fig. 2 | The detailed structure of the dynamic liquid-vapor interface. **(A)** Sequential movie images show fluctuation of the liquid-vapor interface from 44 min 19.23 sec to 44 min 19.53 sec in time step of 0.05 sec. **(B and C)** Magnified sequential images show the fluctuation of two liquid-vapor interfaces from 32 min 21 sec and from 32 min 17 sec, respectively. Figs. were the frames acquired with an OriusSC2006 CCD camera by GATAN Digital Micrograph.

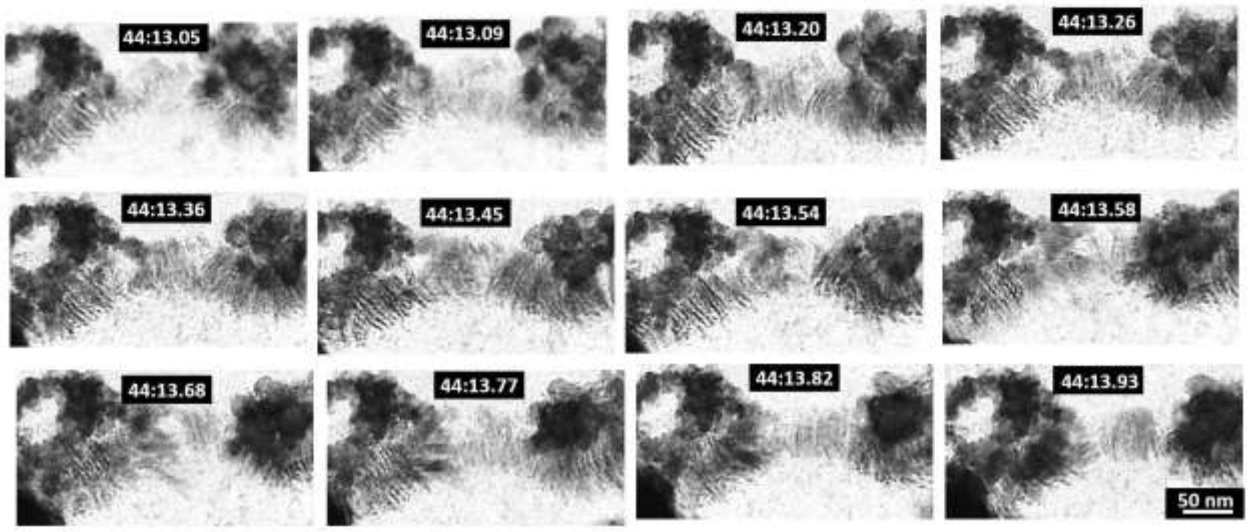

Supplementary Fig. 3 | The detailed structures of the dynamic nanofibers in the liquid-vapor interface. Additional representative zoomed-in images of the liquid-vapor interface, which have fibers containing several nanoparticles (size ~1 nm) with widths of ~1 nm and lengths of ~20–30 nm. Figs. were the frames acquired with an OriusSC2006 CCD camera by GATAN Digital Micrograph.

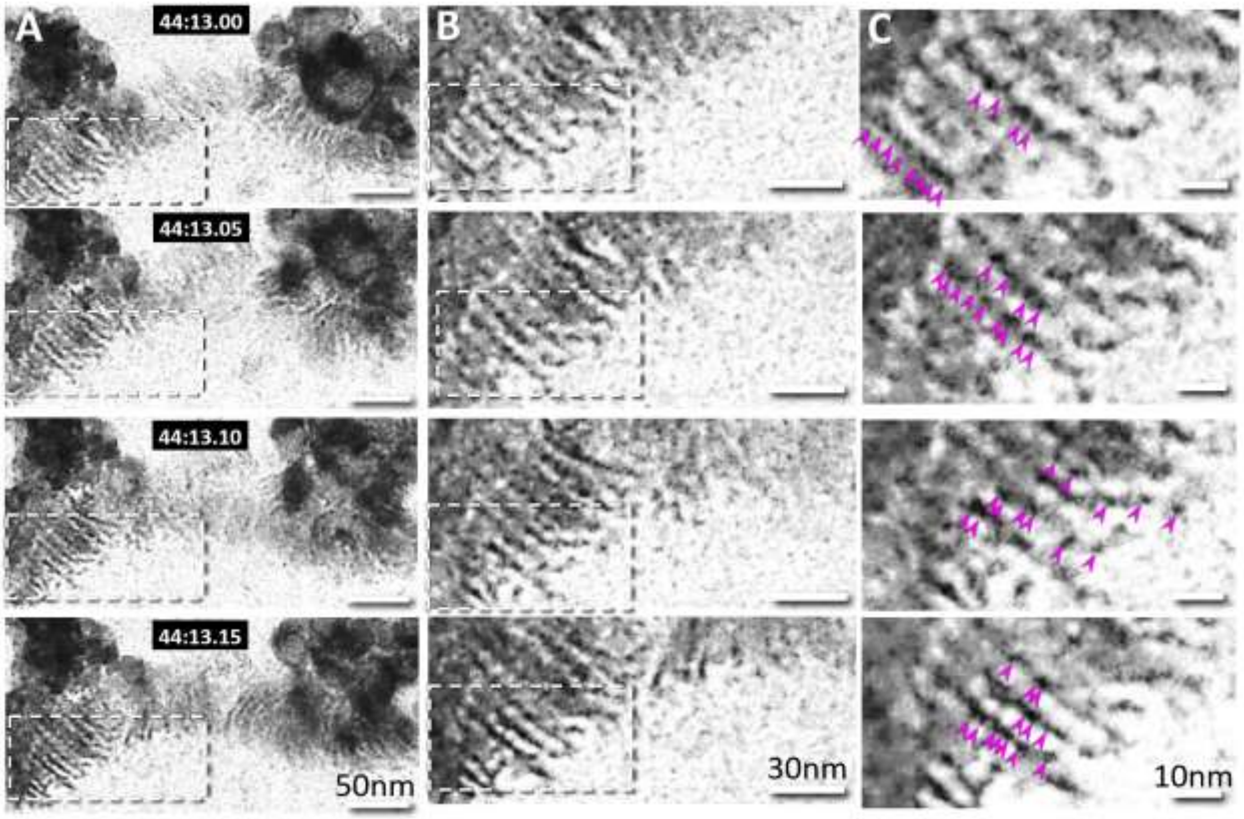

Supplementary Fig. 4 | The ~1 nm tiny nanoparticle imbedded in the dynamic fibers in the liquid-vapor interface. (A) Representative zoomed-in images of a portion of the liquid-vapor interface. **(B)** Further zoomed-in images of the dashed box region in (A). **(C)** Even further zoomed-in images of the dashed boxed region in (B) showed the ~1 nm tiny nanoparticle (indicated by magenta arrows) existed within the fibers (shadow strips) but did not appear in the gap between fibers. Figs. were the frames acquired with an OriusSC2006 CCD camera by GATAN Digital Micrograph.

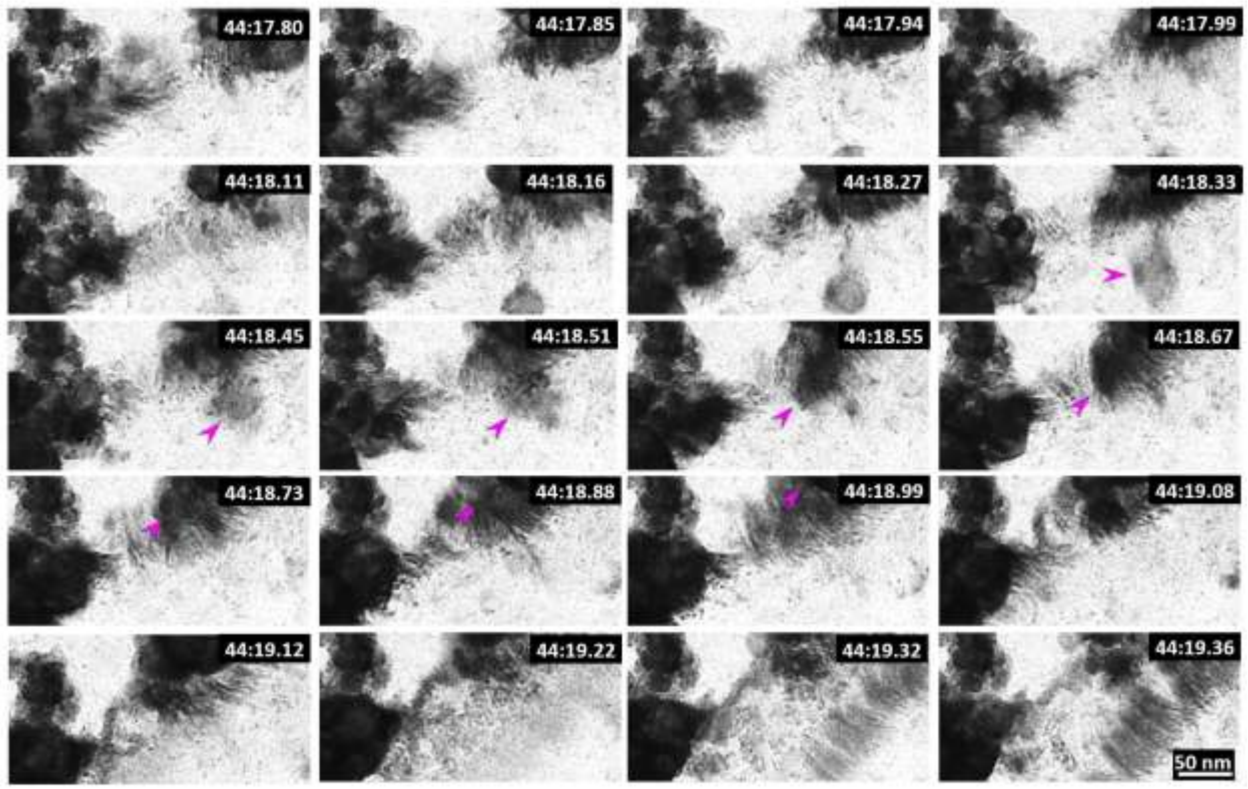

Supplementary Fig. 5 | The detailed structure of a nanoparticle absorption process in the liquid-vapor interface. Representative images of a particle with a diameter of ~30–40 nm (indicated by magenta arrow), which appeared in the horizon, gradually approached the boundary of the liquid-vapor interface, and then was absorbed by the interface, as shown in sequential 2-second snapshots from the video. Figs. were the frames acquired with an OriusSC2006 CCD camera by GATAN Digital Micrograph.

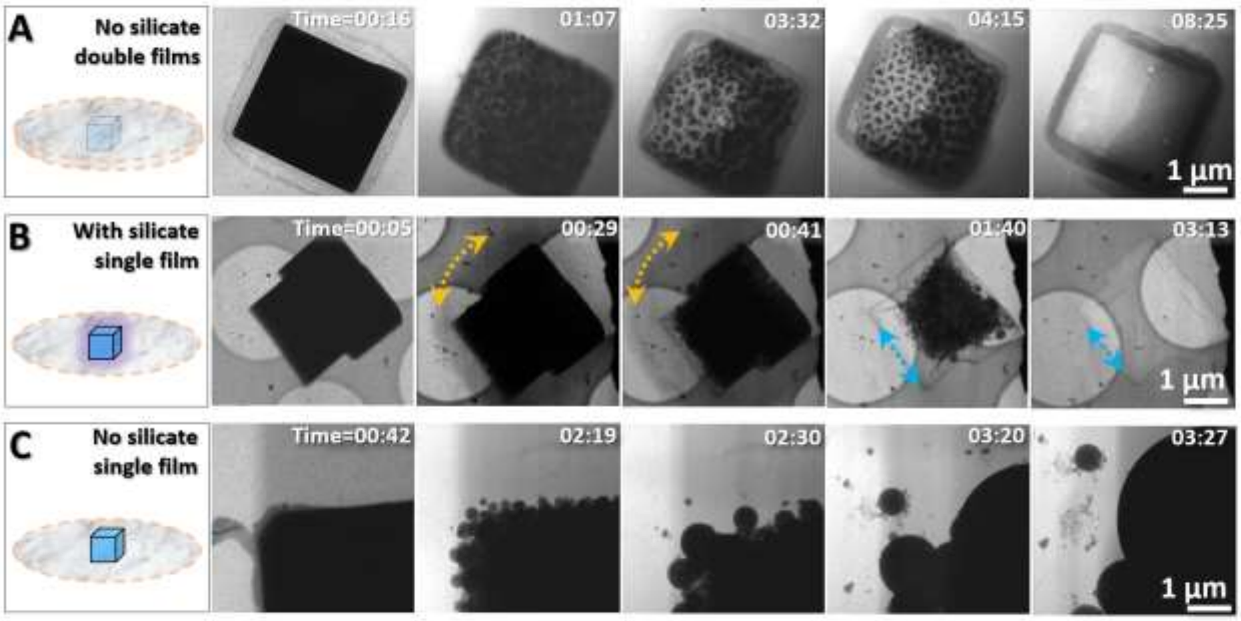

Supplementary Fig. 6 | Evaluation of the role of the liquid-cell/chamber materials. (**A**) A pure NaCl crystal (~99.999%) containing no calcium silicate was sandwiched between two Formvar films, and then melted by electron beam irradiation. (**B**) A NaCl crystal containing calcium silicate was placed on a single film rather than being sandwiched between double films and then melted by electron beam irradiation. The orange dashed arc lines outline the liquid front boundary (the shadowed region). The straight cyan dashed lines outline the leftover "shell" of the crystal. (**C**) A pure crystal (~99.999%) containing no silicate deposited on a single film, and then melted by electron beam irradiation. The roundish particles showed on the surface, no fluid or "shell" was observed. Figs. in the left column were prepared by MICROSOFT POWERPOINT, and Figs. in the rest column were the frames acquired with an OriusSC2006 CCD camera by GATAN Digital Micrograph.

Supplementary Video Legends

Supplementary Video 1. Electron irradiation of NaCl crystals.
Supplementary Video 2. The dynamic structure of liquid-vapor interface
Supplementary Video 3. Brownian motion of nanoparticles in liquid.
Supplementary Video 4. Brownian motion of nanoparticles in vapor.
Supplementary Video 5. Nanoparticle interaction and fusion in liquid.
Supplementary Video 6. "Two-cylinder motor" of spinning liquid within a micro-chamber.
Supplementary Video 7. The detailed structure of the liquid-vapor interface.
